# *SEMA4D*/*PlexinB1* promotes AML progression via activation of *PI3K*/*Akt* signaling

**DOI:** 10.1186/s12967-022-03500-w

**Published:** 2022-07-06

**Authors:** Lu Liu, Lin Yang, Xiaojun Liu, Menghan Liu, Jing Liu, Xuefeng Feng, Ziyuan Nie, Jianmin Luo

**Affiliations:** Department of Hematology, Key Laboratory of Hematology, The Second Hospital of Hebei Medical University, Shijiazhuang, 050000 Hebei China

**Keywords:** Acute myeloid leukemia, *SEMA4D*, *PlexinB1*, *PI3K*/*Akt*, Prognostic

## Abstract

**Background:**

Acute myeloid leukemia (AML) is the most common type of acute leukemia in adults. SEMA4D is a 150 kDa transmembrane protein that belongs to the IV class of the subfamily of semaphorin family. Previous studies have reported that SEMA4D is a multifunctional target in many solid tumors, involving multiple physiological systems, and there are emerging therapies to target these pathways. The role of SEMA4D in AML has not yet been explored.

**Methods:**

The *SEMA4D* expression prolile, clinical data and potential prognostic analysis were acquired via the cBioPortal and GEPIA databases. *SEMA4D* expression was measured using real-time quantitative PCR and western blot. Cell counting kit-8 (CCK8) and flow cytometry were used to evaluate the malignant biological characteristics.

**Results:**

We observed that *SEMA4D* was increased in AML patients and correlated with risk stratification and prognosis. Moreover, *SEMA4D* promotes the proliferation and inhibits apoptosis of AML cells by binding to its receptor, *PlexinB1*, and reduces the sensitivity of AML cells to daunorubicin. In addition, *SEMA4D*/*PlexinB1* promotes the proliferation and survival of AML cells by activating the *PI3K*/*Akt* signaling pathway. VX15/2503, an anti-*SEMA4D* antibody, can inhibit the proliferation of AML cells in xenograft mouse models, thereby inhibiting the development of AML.

**Conclusion:**

*SEMA4D* will serve as a unique predictive biomarker and a possible therapeutic target in AML.

## Background

Acute myeloid leukemia (AML) is a common hematological tumor with extremely high mortality. AML is characterized by aberrant clonal proliferation of immature cells in bone marrow, peripheral blood, and other afflicted tissues, accounting for around 80% of leukemia in adults [1]. Adult AML is currently treated mostly with a combination of chemotherapy and allogeneic hematopoietic stem cell transplantation (allo-HSCT). Despite this, a significant number of patients acquire medication resistance, leading to disease progression. We know that the mortality rate of AML remains high, hence more effective targeted therapy is urgently needed.

Semaphorin 4D (*SEMA4D*) is a member of the semaphorin family [2]. *SEMA4D* is made up of the *SEMA* domain, a cysteine-rich domain, an immunoglobulin domain, a transmembrane domain, and an amino acid terminal signal domain [3, 4]. It is widely expressed in diverse human tissues and organs and is the first signal protein found to have immune regulatory function [5]. It is highly expressed on the surface of most immune cells, including T cells, B cells, natural killer cells and myeloid cells such as monocytes, macrophages and dendritic cells [6–8]. Previous research has linked it to axon guidance, immune system regulation, and nervous system regulation. *SEMA4D* has been reported to be strongly expressed in a variety of solid malignancies, including cutaneous squamous cell carcinoma [9], head and neck squamous cell carcinoma [10], lung cancer [11], breast cancer [12], pancreatic cancer [13], soft tissue sarcoma [14] and others. It can bind to its high affinity receptor *PlexinB1* [15], assist in tumor formation, regulate tumor-associated macrophages, promote tumor angiogenesis, provide nutrients for tumor cells, and further promote the growth, invasion, and migration of tumor cells [16, 17]. However, there has been few studies on the relationship between *SEMA4D* and AML, and its precise mechanism has to be further clarified. Therefore, research into *SEMA4D* and its downstream signaling pathways may provide strategies for the treatment of AML.

Since previous studies have shown *SEMA4D* is a multifunctional target involving several physiological systems, inhibiting its activity could represent a novel therapeutic strategy. VX15/2503 is a humanized IgG4 monoclonal antibody that can specifically bind to *SEMA4D* and prevent *SEMA4D* binding to its receptor [18], which is critical in the physiological process of tumor growth and immune cell regulation [19]. VX15/2503 has been demonstrated in studies to have a synergistic effect with immune checkpoint inhibitors and to boost anti-tumor immunity [19–21]. As a result, the therapeutic medication VX15/2503, which targets *SEMA4D*, may provide a novel cancer treatment method. However, there have been few studies on *SEMA4D* and VX15/2503 in AML, and the particular mechanism is yet unknown.

In this study, we discovered that *SEMA4D* expression was considerably higher in AML patients than in healthy controls, and that it was linked to risk stratification and poor prognosis. AML cell growth was reduced and apoptosis was enhanced when *SEMA4D* was downregulated. We revealed that *SEMA4D* activated the *PI3K*/*Akt* signaling pathway in a *PlexinB1*-dependent manner, thereby promoting the development of AML. We also proved that the anti-*SEMA4D* antibody could reverse the tumor-promoting effect of *SEMA4D*. Our findings suggest that *SEMA4D* could be a new candidate prognostic biomarker as well as a potential therapeutic target for AML.

## Materials and methods

### Database analysis

The Gene Expression Profiling Interactive Analysis (GEPIA) [22] is a popular interactive online server for exploring RNA sequencing data from the TCGA and GTEx projects. We utilized GEPIA to compare *SEMA4D* expression levels in AML patients and healthy people, and we also performed a survival analysis.

The cBio Cancer Genomics Portal (cBioportal) [23] is a comprehensive and user-friendly website that serves as a resource for studying and analyzing TCGA cancer data. For this investigation, we used the cBioportal to collect gene expression profiles and clinical data from AML patients.

### Specimen collection and cell lines

This study was approved by the Second Hospital of Hebei Medical University Ethics Committee. We obtained bone marrow samples from 66 patients with untreated first-ever acute myeloid leukemia and 46 healthy bone marrow donors as a healthy control in this investigation. All AML patients were diagnosed by bone marrow morphology, immunology, cytogenetics, and molecular biology examinations. Human lymphocyte separation solution (HaoYang Biological manufacture Co., Ltd, TianJin, China) was used to isolate bone marrow mononuclear cells (BM-MNCs) from the obtained tissues. The acute myeloid cell lines U937 and Molm-13 were cryopreserved in our laboratory and cultured in Roswell Park Memorial Institute-1640 medium (RPMI-1640; Gibco) containing 10% fetal bovine serum (FBS; Gibco), 100 U/mL penicillin and 100 µg/mL streptomycin. The cell's incubation environment was set to 37 °C in humidified air containing 5% CO_2_.

### RNA extraction and RT-qPCR

Total RNA was isolated from cells using Trizol (Invitrogen, Carlsad, CA, USA). A reverse transcriptase reaction was carried out according to the manufacturer’s instructions using a reverse transcriptase reaction kit (Funeng, Guangzhou, China). Real-time quantitative PCR was carried out with the use of a Real-time quantitative PCR kit (Funeng, Guangzhou, China). The following were the RT-qPCR primer sets: *SEMA4D* specific primers (sense, 5′-GAAGCAGCATGAGGTGTATT-3′; antisense, 5′-GGATGTTAAGTTCAGGTGGTC-3′), *PlexinB1* specific primers (sense, 5′-ATTCACTCCCAATGGCACG-3′; antisense, 5′-GGCACTCATCAGGCATCACA-3′), GAPDH specific primers (sense, 5′-CCTCTGACTTCAACA GCGACAC-3′; antisense, 5′-TGGTCCAGGGGTCTTACTCC-3′).

### Western blot

Total proteins were extracted from cells with RIPA Lysis Buffer (Solarbio Technology Co., Ltd., Beijing, China). Protein concentration was determined using the Bicinchoninic Acid Kit (Boster Biological Company, Ltd., Wuhan, China). An analytical 10% SDS-PAGE was performed, and 30 μg of protein from each was analyzed. The proteins were then transferred to polyvinylidene difluoride membranes, which were then blocked for 1 h in 5 percent nonfat milk TBST. The protein bands were then incubated with the appropriate primary antibodies overnight at 4 °C before being incubated with the secondary antibodies for 1 h. The bands were visualized using the BioSpectrum Imaging System (UVP, LLC, Upland, CA, USA). Primary antibodies were as follows: *SEMA4D* (1:600; ABclonal Technology, Wuhan, China; A10136), *Bcl-2* (1:600; Boster Biological Company, Ltd., Wuhan, China; BM0200), *Bax* (1:600; Boster Biological Company, Ltd., Wuhan, China; A00183), *Cleaved-Caspase3* (1:1000; Abcam, CA, USA;ab32042), p-*PI3K* (1:1000; Cell Signaling Technology, USA; #4228), *PI3K* (1:1000; Cell Signaling Technology, USA; #4292), p-*Akt* (1:1000; Cell Signaling Technology, USA; #9271), *Akt* (1:1000; Cell Signaling Technology, USA; #9272), *PlexinB1* (1:600; Proteintech Group, Inc., USA; 23795-1-AP), *β-ACTIN* (1:8000; Abways Technology, New York, NY, USA; AB0035) and goat-anti-rabbit (1:10,000, Boster Biological Company, Ltd., Wuhan, China).

### Cell transfection

U937 and Molm-13 cells were grown to a log phase in RPMI-1640 media containing 10% fetal bovine serum (FBS) before transfection. Lentiviruses containing shRNA-*SEMA4D* or overexpression of *SEMA4D* plasmid were constructed by the Shanghai Genechem Co., Ltd. U937 cells cultured in 24-well plates were infected with virus at a multiplicity of infection (MOI) of 30 and Molm-13 cells were infected with virus at a MOI of 50. The cells were then maintained for 12–16 h. After 48 h of transfection, the cells were treated with 2 µg/mL puromycin to create a stably transfected cell line. To mute the expression of *PlexinB1*, small interfering RNAs (*PlexinB1*-siRNA: 5′-AGAAGAUGCAGCUGGGCUATT-3′; Si-control: 5′-UUCUCCGAACGUGUCACGUTT-3′) were generated by Anhui General Biosystems Co., Ltd (Anhui, China). SiRNA transfections were carried out according to the manufacturer’s instructions using the Advanced DNA RNA Transfection Reagent (Zeta Life, United States, AD600025).

### Cell growth and proliferation assays

Cells were inoculated in 96-wells plate with 1 × 10^5^ cells per well. They were cultured for a total of 0 h, 24 h, 48 h, 72 h, and 96 h. Cells were grown for 1–4 h at 37 °C in 100 μL culture medium containing 10 μL Cell Counting Kit-8 reagent (Beibo Biological Reagent Co., Shanghai, China). Microplate Reader (BioTek, Winooski, VT, USA) was used to measure the absorbance of each well at 450 nm. For the colony formation assay, 3 × 10^3^ cells were cultured in each well of six well plates for 2 weeks at 37 °C with 5% CO_2_ saturated humidity in the methylcellulose medium. The methylcellulose medium was mixed with 2 g methylcellulose, 50 mL ultrapure water and 50 mL 2xPRMI 1640 (Gibco; 31800022), which was then supplemented with 20% FBS and 1% antibiotics (100 U/mL streptomycin and 100 mg/mL penicillin). The numbers of colonies containing more than 50 cells were counted.

### Apoptosis assay

The cells were collected by centrifugation and washed in PBS. An Annexin V-APC/PI apoptosis kit was used to evaluate cell apoptosis (70-AP101-100, MULTISCIENCES BIOTECH CO., Hangzhou, China). The cells required for different experiments were mixed with 5 µL Annexin V/APC and 10 µL propidium iodide (PI) based on the manufacturer’s instructions. The cells were then incubated in the dark for 5 min. They were analyzed with a FC500 flow cytometer (Beckman Coulter). Kaluza software (Beckman Coulter) was used to analyze the data.

### Drug treatment

The CCK-8 assay is used to measure chemosensitivity to daunorubicin (HY-13062; MedChemExpress). Cells were plated in 96-well plates and treated with Daunorubicin at concentrations ranging from 1.25 to 640 nmol/L for MOLM-13 and 10 to 1280 nmol/L for U937, with at least three technical replicates per concentration per cell line. After 48 h of incubation at 37 °C in a 5% CO_2_ environment, 10 μL of CCK-8 solution is added to each well. To get the IC50 values, a nonlinear fit of log (inhibitor) versus normalized response was performed in GraphPad Prism v8.0.2. Furthermore, U937 cells were treated with daunorubicin at a concentration of 80 nmol/L and Molm-13 cells were subjected at a concentration of 10 nmol/L for an apoptotic experiment. VX15/2503, anti-human *SEMA4D* antibody was synthesized by AtaGenix Laboratories (ATAD00383, WuHan, China). Cells were grown in 6-well plates and exposed at doses of 20 µg/mL and then carried out subsequent experiments.

### Animal experiment

Eight female severe combined immunodeficient (SCID) nude mice (15–20 g, 5–6 weeks’ old) were implanted subcutaneously dorsally with U937 cells (1 × 10^7^) suspended in 200 µL PBS. The mice were kept at an ambient temperature of 18–22 °C and a relative humidity of 50–60% without pathogens. The tumor size and body weight were dynamically observed every day. When the tumor size reached 30 mm^3^, the mice were randomly divided into an experimental group or a control group (*n* = 4 mice/group). Anti-*SEMA4D* antibody VX15/2053 (1.5 mg/kg/d) was given to the experimental group, while the control group received normal saline. Antibody was given by subcutaneous injection around the tumor region. During the experiment, changes in tumor volume were measured with calipers every 2 days. The nude mice were sacrificed on day 14 after drug treatment. All animal experimental protocols were approved by the Animal Experimental Committee of Hebei Medical University and implemented in accordance with the Guide to Animal Experiment.

### Immunohistochemistry

The small tissue of xenografts removed from the animal body was immediately fixed in pre-prepared liquid fixative, 10% formalin, and then embedded in paraffin. The embedded paraffin sections were routinely dehydrated and sectioned. The tissue sections were incubated overnight with the primary antibody at 4 °C and for 30 min with the secondary antibody at room temperature. Sections washed with PBS were incubated with DAB developer for 3–10 min at room temperature.

### Statistical analysis

Statistical Package for the Social Sciences (SPSS, Version 26.0) software was used for statistical analysis. Student’s t-test, Mann–Whitney test, Pearson’s chi-square test and Fisher’s exact test were used to compare different groups. P values less than 0.05 were considered statistically significant (*P < 0.05, **P < 0.01, ***P < 0.001). Experiments were repeated at least three times. We visualized statistical results using GraphPad Prism (Version 8.0.2).

## Results

### Expression level of *SEMA4D* in AML

**Fig. 1:**
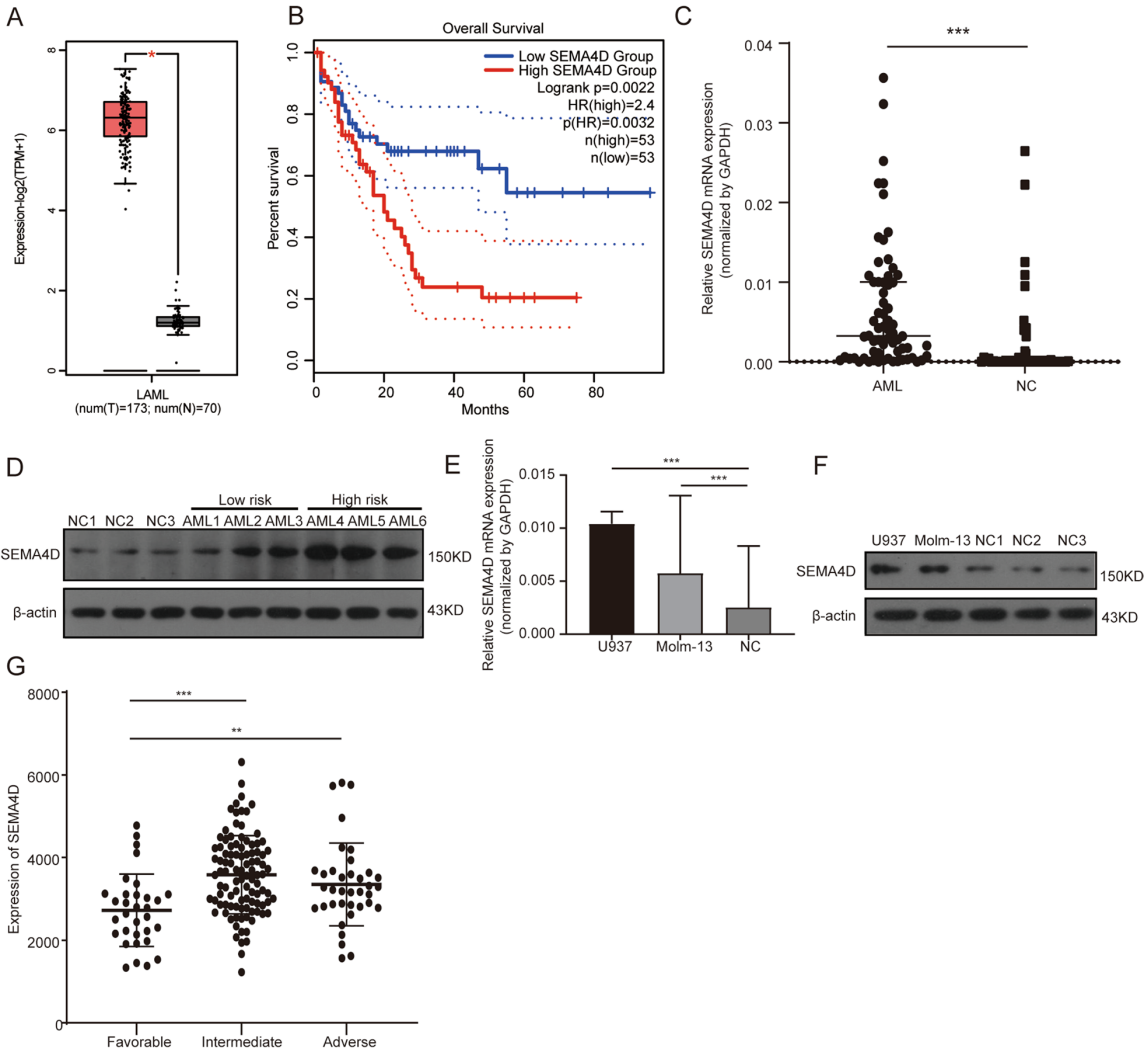
Expression level of SEMA4D in AML. **A** Expression level of SEMA4D in LAML compared with healthy control in GEPIA Database. **B** The OS of patients with LAML in GEPIA Database. **C** qRT-PCR was used to detect SEMA4D mRNA level in BM-MNCs of AML patients and healthy donors. **D** Western blot was used to detect SEMA4D protein levels in BM-MNCs of AML patients and healthy donors. **E** qRT-PCR was used to detect SEMA4D mRNA level in U937 and Molm-13. **F** Western blot was used to detect SEMA4D protein levels in U937 and Molm-13. Results of densitometry analysis of relative expression levels after normalization to loading control β-actin are presented. **G** Expression of SEMA4D in LAML based on risk status in TCGA database. Data with statistical significance are as indicated, *P < 0.05, **P < 0.01, ***P < 0.001, *ns* not significant

Firstly, we examined the expression of *SEMA4D* in AML in the GEPIA database to see whether the expression level of *SEMA4D* was related to prognosis of AML. *SEMA4D* was found to be highly expressed in AML compared to normal controls (Fig. 1A), and patients with high *SEMA4D* expression had a poor prognosis (Fig. 1B). These findings provided us with useful information that *SEMA4D* up-regulation affects AML prognosis. We further collected 66 AML patients and 46 normal subjects in our hospital for qPCR and WB validation. As shown in Fig. 1C, D, *SEMA4D* expression levels at the mRNA and protein levels were higher than in the normal control group. In addition, the expression of *SEMA4D* was also markedly upregulated in the AML cell lines U937 and Molm-13 compared with the control group (Fig. 1E, F).

Since the TCGA database contains a sufficient number of samples, we obtained *SEMA4D* expression data and the clinical data of the TCGA dataset from cBioPortal to study the relationship between *SEMA4D* expression and AML clinical and laboratory characteristics. A total of 173 cases (Table 1) with both gene expression and clinical data were available for subsequent analysis. Patients were divided into two groups: those with high expression of *SEMA4D* (*n* = 78) and those with low expression of *SEMA4D* (*n* = 95) based on the mean value of *SEMA4D* expression. As indicated in Table 1, we examined *SEMA4D* expression levels in different risk categories and discovered that *SEMA4D* expression levels in the low-risk group were significantly lower than those in the intermediate-risk group and adverse-risk group (Fig. 1G, Intermediate vs*.* Favorable: *P* < 0.001; Adverse vs. Favorable: *P* = 0.002). There was no statistically significant difference between the two groups of patients in terms of gender, age, white blood cell count, peripheral blood blast, or bone marrow blast (Table 1). Our findings indicate that *SEMA4D* expression is upregulated in human acute myeloid leukemia and corresponds with prognosis and risk stratification. This suggests to us that *SEMA4D* may play a potential role in AML.

**Table 1:** Relationships between SEMA4D mRNA expression and baseline characteristics of patients with AML from cBioportal

Clinical characteristics	Total	SEMA4D low (n = 95)	SEMA4D high (n = 78)	P-value
Age				
< 60	91	53 [58.2%]	38 [41.8%]	0.354
> 60	82	42 [51.2%]	40 [48.8%]	
Gender				
Male	92	53 [57.6%]	39 [42.4%]	0.448
Female	81	42 [51.9%]	39 [48.1%]	
Risk vs. favorable				
Favorable	32	27 [81.3%]	5 [18.8%]	
Intermediate	101	44 [40.6%]	57 [59.4%]	0.000
Adverse	37	22 [48.6%]	15 [51.4%]	0.002
Missing	3	2 [66.7%]	1 [33.3%]	
Lab examinations				
WBC		22.9 (3.4–57.1)	14 (3.05–51.95)	0.519
BM		72 (54–86)	75 (50.75–85.25)	0.905
PB		45 (7.25–72)	27 (7.75–58.75)	0.123

### *SEMA4D* promotes the proliferation and survival of AML cells and affects chemotherapy sensitivity

**Fig. 2:**
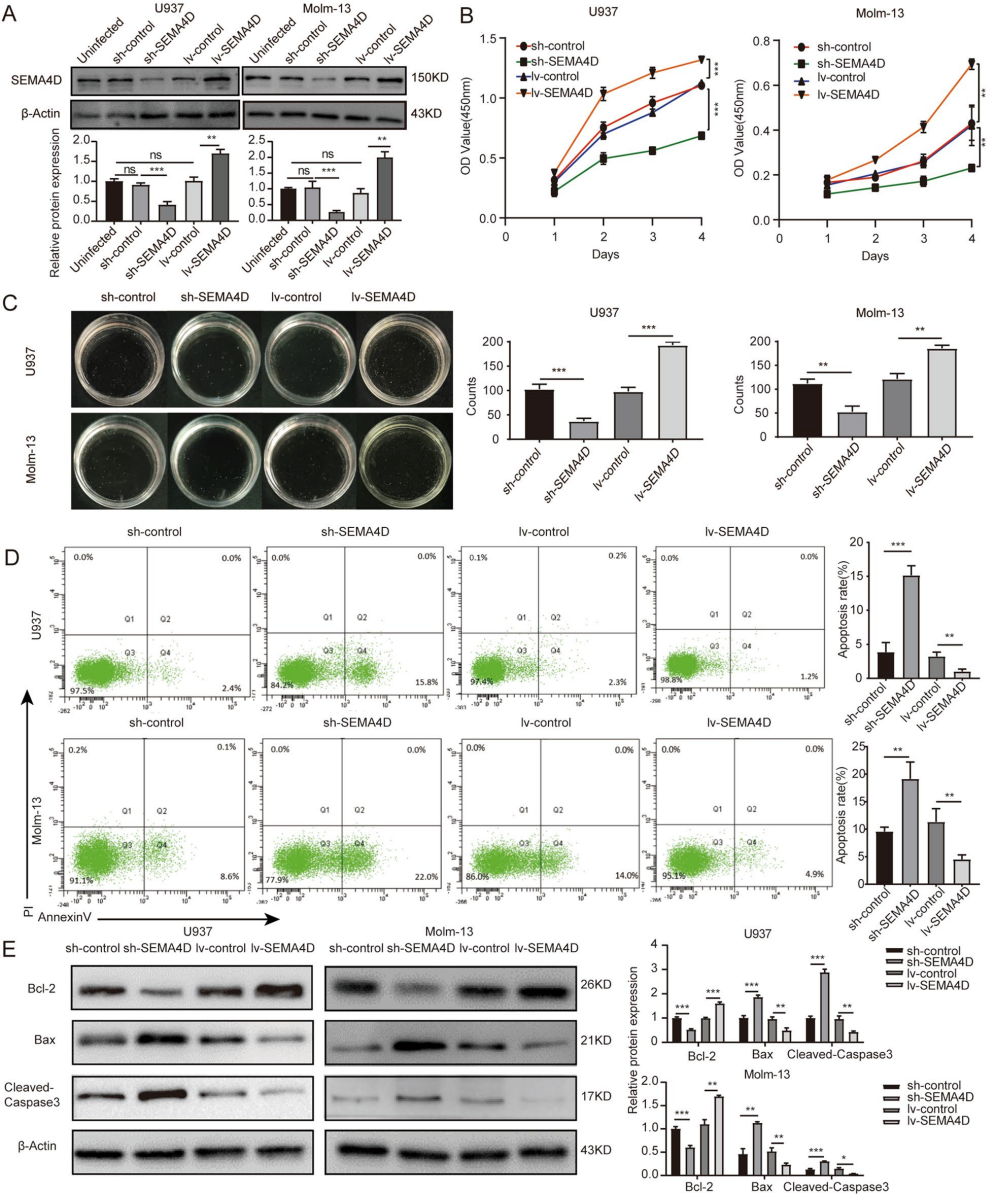
SEMA4D promotes proliferation and inhibits apoptosis of AML cells. **A** Western blot was used to detect SEMA4D protein level when U937 and Molm-13 cells were transfected with stably knocking down or overexpressing SEMA4D lentivirus. **B** CCK-8 analysis of U937 and Molm-13 cells transfected with lentivirus targeting SEMA4D or control. **C** Colony formation assay of U937 and Molm-13 cells transfected with lentivirus targeting SEMA4D or control. **D** Cell apoptosis rate of U937 and Molm-13 cells transfected with lentivirus targeting SEMA4D or control was detected by flow cytometry using Annexin V-APC/PI staining. **E** Western blotting analysis was used to determine the expression of apoptosis-related proteins (Bcl-2, Bax, and cleaved-caspase3) in U937 and Molm-13 cells transfected with lentivirus targeting SEMA4D or control. Results of densitometry analysis of relative expression levels after normalization to loading control β-actin are presented. Data with statistical significance are as indicated, *P < 0.05, **P < 0.01, ***P < 0.001, *ns* not significant

*SEMA4D* is highly expressed in AML cells, implying that *SEMA4D* may be related to the biological behavior of cells. To investigate the biological function of *SEMA4D* in AML, we constructed U937 and Molm-13 cells stably knocking down or overexpressing *SEMA4D* by lentiviral infection and further confirmed the expression of *SEMA4D* by western blot (Fig. 2A). The effect of *SEMA4D* on the proliferation ability of U937 and MOLM-13 cells was next investigated utilizing the CCK-8 test and the colony formation assay. As shown in Fig. 2B, *SEMA4D* downregulation considerably reduced cell proliferation, whereas *SEMA4D* overexpression greatly increased cell proliferation when compared to their respective controls. Furthermore, colony-formation experiments demonstrated that clonogenic potential was decreased after *SEMA4D* knockdown but enhanced after *SEMA4D* overexpression (Fig. 2C).

Flow cytometry results revealed that *SEMA4D* depletion could enhance apoptosis in U937 and Molm-13 cells, but *SEMA4D* overexpression could reduce apoptosis (Fig. 2D). Furthermore, when *SEMA4D* was knocked down, the levels of *Bax* and cleaved-caspase3 protein in U937 and Molm-13 cells were considerably increased, while *Bcl-2* protein was downregulated. However, the tendency was reversed when *SEMA4D* was overexpressed (Fig. 2E). These findings suggest that *SEMA4D* could shield leukemia cells from apoptosis.

**Fig. 3:**
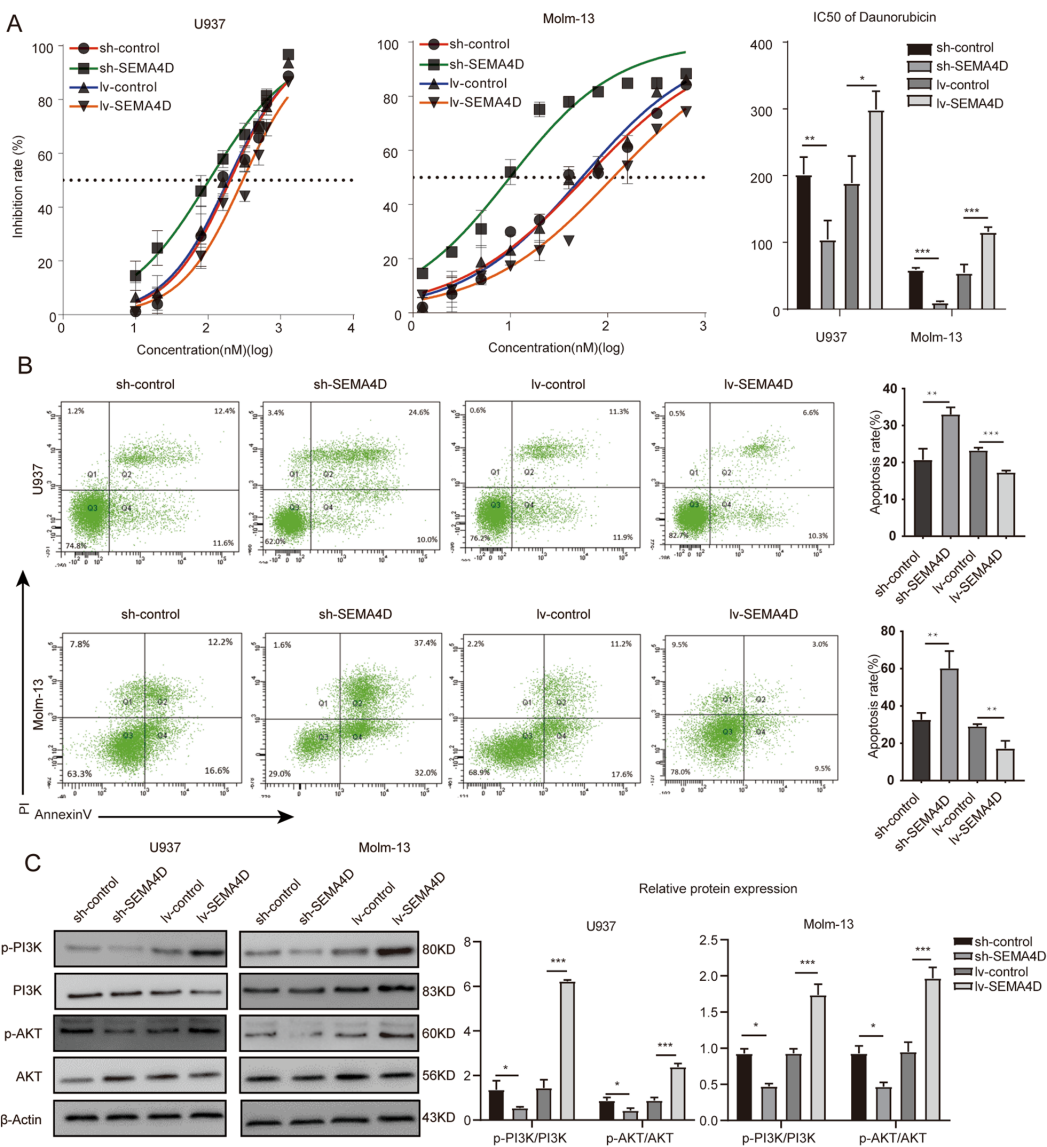
SEMA4D affects chemotherapy sensitivity of daunorubicin and mediates PI3K/Akt phosphorylation in AML cells. **A** IC50 curves of Daunorubicin in U937 and Molm-13 cells transfected with lentivirus targeting SEMA4D or control. Transduced AML cells were treated with daunorubicin for 48 h and then measured by CCK-8 analysis. **B** Cell apoptosis rate of U937 and Molm-13 cells transfected with lentivirus targeting SEMA4D or control after Daunorubicin drug treatment was detected by flow cytometry using Annexin V-APC /PI staining. **C** Western blotting analysis was used to determine the expression of p-PI3K, PI3K, p-Akt, Akt in U937 and Molm-13 cells transfected with lentivirus targeting SEMA4D or control. Results of densitometry analysis of relative expression levels after normalization to loading control β-actin are presented. Data with statistical significance are as indicated, *P < 0.05, **P < 0.01, ***P < 0.001, *ns* not significant

In addition, a significant decrease of IC50 in U937 and MOLM-13 cells upon deletion of *SEMA4D* and an increase of IC50 after overexpression of *SEMA4D* validated the effect of *SEMA4D* on daunorubicin sensitivity in AML cells (Fig. 3A). U937 cells were treated with daunorubicin at a concentration of 80 nmol/L and Molm-13 cells were exposed at a dose of 10 nmol/L for apoptosis assay. We discovered that the rate of apoptosis was raised in cells that had *SEMA4D* knocked down and reduced in cells that had *SEMA4D* overexpressed after treatment with the same concentration of daunorubicin (Fig. 3B). Taken together, these results revealed that *SEMA4D* promotes the proliferation and survival of AML cells and affects chemotherapy sensitivity.

### *SEMA4D* mediates *PI3K/Akt* phosphorylation in AML cells

The *PI3K*/*Akt* signaling system is a critical intracellular signaling pathway that is involved in cell proliferation, apoptosis, differentiation, and metabolism, and is widely employed in clinical illness mechanistic studies. The *PI3K*/*Akt* pathway is key for hematopoietic cells, and it is believed to be constitutively activated in 60% of AML patients, with this activation correlating to a reduced average survival rate [24]. *SEMA4D* has been shown to cause tremendous proliferation and invasion by activating the *PI3K*/*Akt* signaling pathway [13, 25–30]. We investigated the phosphorylation levels of *PI3K* and *Akt* in U937 and Molm-13 cells to better understand the effect of *SEMA4D* on *PI3K* and *Akt* phosphorylation in acute myeloid leukemia. The results showed that *SEMA4D* overexpression elevated the phosphorylation level of *PI3K* and *Akt*, while *SEMA4D* knockdown decreased the phosphorylation of *PI3K* and *Akt* in both U937 and Molm-13 cells (Fig. 3C). Collectively, the results indicated that *SEMA4D* could mediate the activation of the *PI3K*/*Akt* pathway in AML cells.

### *SEMA4D* functions through its receptor PlexinB1

**Fig. 4:**
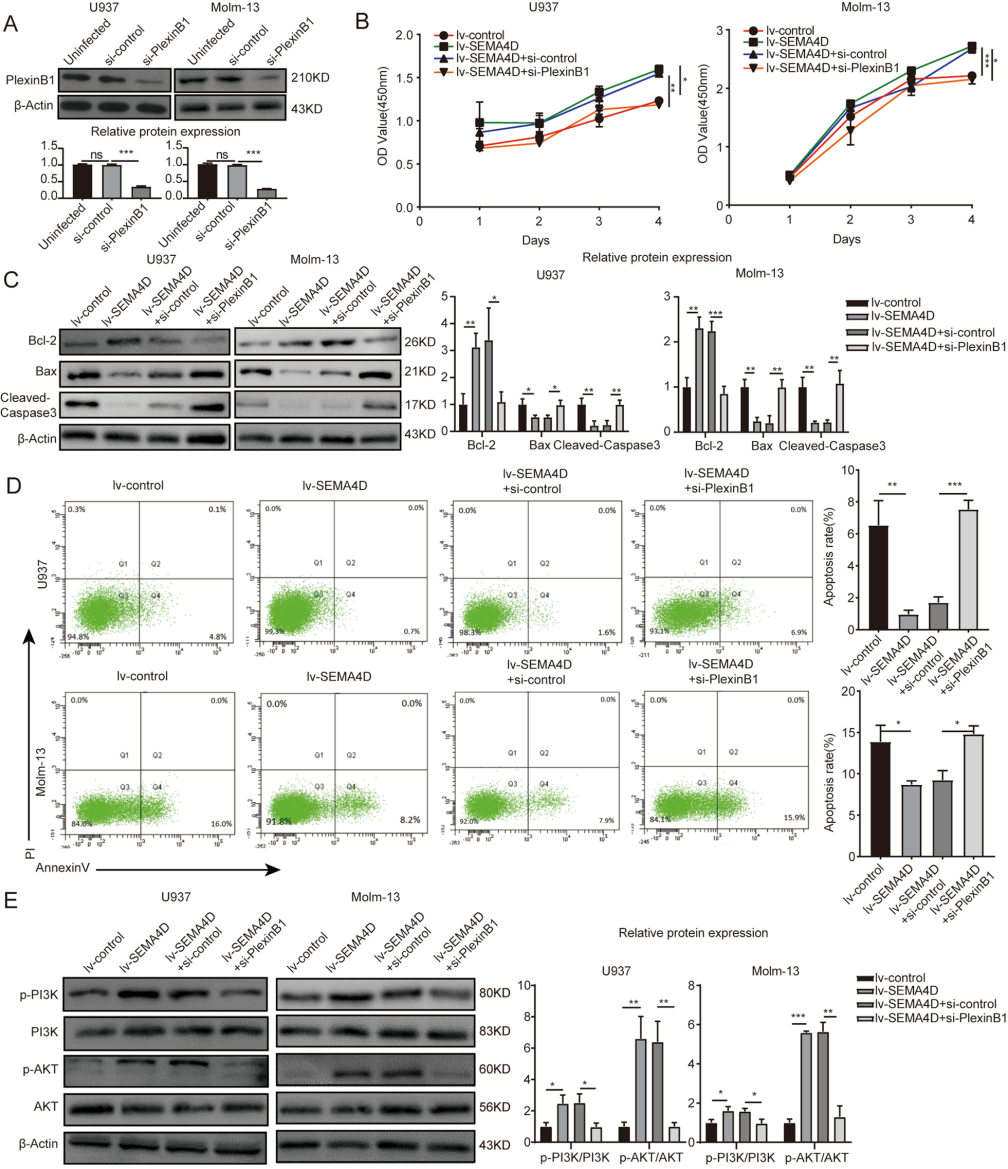
SEMA4D functions through its receptor PlexinB1. **A** Western blot was used to detect PlexinB1 protein level when U937 and Molm-13 cells were transfected with siRNA-PlexinB1 or siRNA-control. **B** CCK-8 analysis of U937 and Molm-13 cells transfected with lentivirus targeting SEMA4D when PlexinB1 was knocked down or not. **C** Western blotting analysis was used to determine the expression of apoptosis-related proteins (Bcl-2, Bax, and cleaved-caspase3) in U937 and Molm-13 cells transfected with lentivirus targeting SEMA4D when PlexinB1 was knocked down or not. Results of densitometry analysis of relative expression levels after normalization to loading control β-actin are presented. **D** Cell apoptosis rate of U937 and Molm-13 cells transfected with lentivirus targeting SEMA4D when PlexinB1 was knocked down or not was detected by flow cytometry using Annexin V-APC/PI staining. **E** Western blotting analysis was used to determine the expression of p-PI3K, PI3K, p-Akt, Akt in U937 and Molm-13 cells transfected with lentivirus targeting SEMA4D when PlexinB1 was knocked down or not. Results of densitometry analysis of relative expression levels after normalization to loading control β-actin are presented. Data with statistical significance are as indicated, *P < 0.05, **P < 0.01, ***P < 0.001, *ns* not significant

*PlexinB1* is a high-affinity *SEMA4D* receptor that can activate downstream signaling in tumor cells after binding to *SEMA4D*, influencing their biological activity [31, 32]. The combination of *SEMA4D* and *PlexinB1* can also activate the c-*Met* tyrosine kinase, leading to tumor growth [33]. *PlexinB1* was efficiently inhibited by transfecting *PlexinB1*-specific siRNA (Fig. 4A). AML cells were transfected with lentiviral over-expressing *SEMA4D* and si-*PlexinB1* to further verify the effects of *SEMA4D* and the receptor *PlexinB1* on cells in acute myeloid leukemia. When *PlexinB1* was knocked down, the pro-proliferative (Fig. 4B) and anti-apoptotic (Fig. 4C, D) effects of overexpression of *SEMA4D* were dramatically decreased. *PlexinB1* knockdown also reduced the phosphorylation of *PI3K*/*Akt* in U937 and Molm-13 cells caused by *SEMA4D* (Fig. 4E). Taken together, these findings demonstrated that *SEMA4D* functions through its receptor *PlexinB1*.

### Anti-*SEMA4D* antibody can inhibit the survival of AML cell lines in vivo and in vitro

**Fig. 5:**
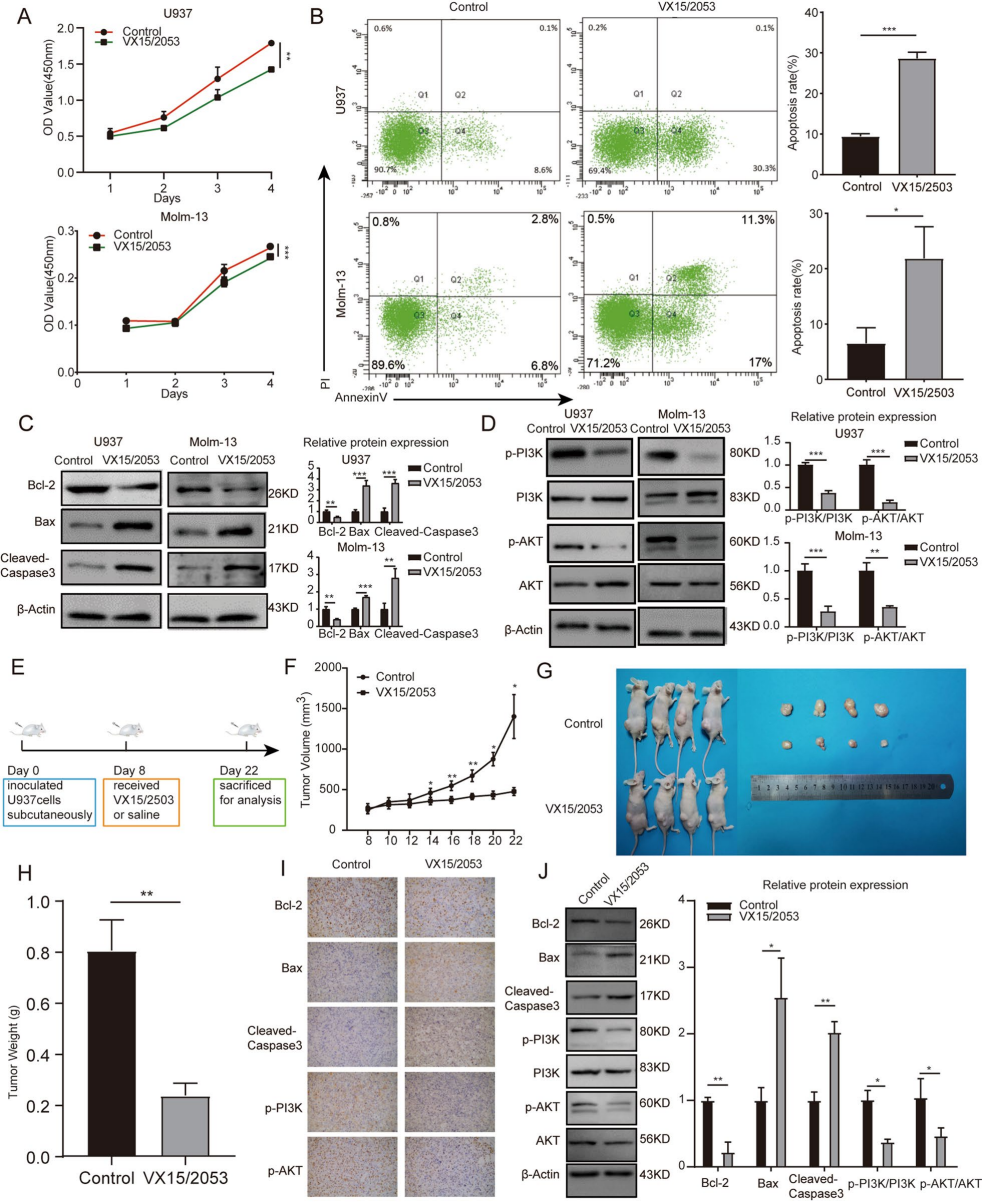
Anti-SEMA4D antibody can inhibit the survival of AML cell lines in vivo and in vitro. **A** CCK-8 analysis of U937 and Molm-13 cells treated with VX15/2503 or not. **B** Cell apoptosis rate of U937 and Molm-13 cells treated with VX15/2503 or not was detected by flow cytometry using Annexin V-APC/PI staining. **C** Western blotting analysis was used to determine the expression of apoptosis-related proteins (Bcl-2, Bax, and cleaved-caspase3) in U937 and Molm-13 cells treated with VX15/2503 or not. Results of densitometry analysis of relative expression levels after normalization to loading control β-actin are presented. **D** Western blotting analysis was used to determine the expression of p-PI3K, PI3K, p-Akt, Akt in U937 and Molm-13 cells treated with VX15/2503 or not. Results of densitometry analysis of relative expression levels after normalization to loading control β-actin are presented. **E** Schematic outline of the mouse model delineating this experiment. **F** Nude mice were subcutaneously inoculated with U937 cells to establish AML xenograft tumors and treated with VX15/2503. Volumes of tumors were monitored by direct measurement. **G** Tumor size of xenograft mice in two groups. **H** Weights of tumors of xenograft mice in two groups. **I** Immunohistochemistry stain was used to measure the expression of Bcl-2, Bax, cleaved-caspase3, p-PI3K, p-Akt in xenograft tumors. **J** Western blotting analysis was used to determine the expression of Bcl-2, Bax, cleaved-caspase3, p-PI3K, PI3K, p-Akt, Akt in xenograft tumors. Results of densitometry analysis of relative expression levels after normalization to loading control β-actin are presented. Data with statistical significance are as indicated, *P < 0.05, **P < 0.01, ***P < 0.001, *ns* not significant

Since *SEMA4D*/*PlexinB1* has been linked to the survival of AML cells, we used the anti-*SEMA4D* antibody VX15/2503 to treat U937 and Molm-13 cells. VX15/2503 suppressed U937 and Molm-13 cell proliferation (Fig. 5A), increased apoptosis rate (Fig. 5B), and elevated the expression of apoptosis protein (Fig. 5C). In addition, in the VX15/2503 group, phosphorylation of *PI3K* and *Akt* was reduced (Fig. 5D). The effect of VX15/2503 on AML in vivo was explored through using nude mice xenograft model. The nude mice were implanted with U937 cells subcutaneously (Fig. 5E). The volume and weight of the tumor were significantly lower in the anti-*SEMA4D* antibody (1.5 mg/kg) treated mice xenograft model than in the saline group (Fig. 5F–H). In addition, immunohistochemistry (Fig. 5I) and western blot analysis were used to detect the protein levels of *Bcl-2*, *Bax*, *Cleaved-Caspase3*, p-*PI3K*/*PI3K*, and p-*Akt*/*Akt* (Fig. 5J). When compared to the saline group, the *Bcl-2*, p-*PI3K*, and p-*Akt* protein levels in the VX15/2503 group decreased while *Bax* and *Cleaved-Caspase3* increased. The results proved that VX15/2503 inhibited the proliferation and survival of AML cells and decreased the phosphorylation of *PI3K*/*Akt* both in vivo and in vitro. Therefore, we confirmed that VX15/2503, a drug targeting *SEMA4D*, can inhibit the survival of AML cell lines in vivo and in vitro.

## Discussion

*SEMA4D* is a transmembrane protein of 150 kDa that belongs to the IV class of the semaphorin subfamily. Previous research has consistently demonstrated that *SEMA4D* has a role in immunosuppression [34], carcinogenesis, and progression [9, 35, 36]. According to Zuazo-Gaztelu and colleagues, *SEMA4D* expression increased with tumor progression in pancreatic neuroendocrine cancer, and anti-*SEMA4D* antibody reduces tumor growth and tends to lengthen mouse lifespan [37]. A prior investigation into hematologic cancers found that *SEMA4D* is present in virtually all CLL cells and is crucial in sustaining CLL cell survival and proliferation [36]. We combined *SEMA4D* expression data in AML from the TCGA database and further validated *SEMA4D* expression levels in AML patients from our center. Similar to earlier publications, *SEMA4D* was discovered to be highly expressed in AML, and its expression was revealed to be directly connected to risk stratification. Our data further elaborated that reducing the expression of *SEMA4D* induces apoptosis and limits the cell growth of AML. Furthermore, we discovered that inhibiting *SEMA4D* might increase the sensitivity to daunorubicin. As a result, the foregoing findings may imply that *SEMA4D* is an essential regulatory factor for cell proliferation and may influence chemotherapy effectiveness in AML.

In addition to the possible use of *SEMA4D* as a biomarker for AML, a potential mechanism of *SEMA4D* was identified. The *PI3K*/*Akt* pathway is essential for hematopoietic cells, and affects vital processes such as proliferation, differentiation, and survival [24]. *SEMA4D* has been shown to be highly expressed in childhood acute lymphoblastic leukemia and promotes ALL development by activating *PI3K*/*Akt* and *ERK* signaling pathways [27]. Moriarity et al. showed *SEMA4D* is associated with osteosarcoma development, and was found to be enriched in the *PI3K*-*Akt*-*mTOR* signaling pathway [38]. We speculate that *SEMA4D* may also affect the proliferation and survival of cells by affecting the *PI3K*/*Akt* pathway in acute myeloid leukemia. We now show SEMA4D also affects the proliferation and survival of cells in AML by similar mechanisms.

Furthermore, *PlexinB1* has been shown to be a readily accessible receptor for *SEMA4D* inside the immune system. Interactions between *SEMA4D* and *PlexinB1* may enhance the proliferation and survival of malignant cells, implying that *SEMA4D* expression is related to the malignant process in patients [36]. Ikeya et al. validated that the combined expression of *SEMA4D* and *PlexinB1* predicts disease recurrence in colorectal cancer [39]. *PlexinB1* has been demonstrated to enhance resistance to androgen receptor pathway inhibition in the treatment of prostate cancer [40]. Therefore, we knocked down the expression of *PlexinB1* on the basis of overexpression of *SEMA4D*, and the results showed that the role played by *SEMA4D* was greatly weakened, thus demonstrating that *SEMA4D* promotes the phosphorylation of *PI3K*/*Akt* in AML in a *PlexinB1*-dependent manner, thereby promoting the proliferation and survival of AML cells.

By binding to the homogenous region of *SEMA4D* and spatially interfering with the binding site of *PlexinB1* [41], VX15/2503 disrupts the interaction between *SEMA4D* and its receptor. The safety study of VX15/2503 showed that the antibody had no clinical or toxicological consequences [42]. The antibody is currently being tested in clinical trials and could be a potential therapeutic method for the treatment of a variety of cancers as well as several autoimmune illnesses. VX15/2503 was found to be safe and well tolerated in adult patients with advanced solid tumors in the first clinical trial, with 45 percent of patients showing no disease progression for at least 8 weeks [43]. As a result, we used anti-*SEMA4D* antibody to disrupt *SEMA4D* binding to its receptor in U937 and Molm-13 cells, and discovered that it may decrease the downstream physiological consequences. Furthermore, we found that anti-*SEMA4D* antibody suppresses tumorigenic potential both in vitro and in vivo in this investigation. These findings demonstrated that therapy with anti-*SEMA4D* antibody had an anti-tumor impact and backed up our hypothesis.

Interestingly, Escuredo found that 81% of renal cell carcinoma lost the *PlexinB1* expression [44]; although Zuazo-Gaztelu et al. found that the treatment of anti-*SEMA4D* antibody could decrease tumor proliferative activity and extend the life span of mice, an unexpected increase in tumor metastasis was observed, and they found that the number of macrophages with positive *SEMA4D* expression increased significantly after treatment and may promote cancer progression through increased release of *SDF1*/*CXCL12* [37]. Some scholars speculate that this seemingly contradictory response caused by the signal of *SEMA4D*/*PlexinB1* may be due to the coexistence of stimulation and inhibition domains in the *PlexinB1* cytoplasmic domain. They speculate that the “intramolecular” preferential *PlexinB1* signaling pathway in cancer can constitute a reasonable explanation to reconcile the contradictory clinical results of *PlexinB1* in tumor progression [32]. However, the precise mechanism behind these findings remains to be elucidated. In addition, our experiment may require further study in the leukemia mouse model, and further study on the of the role and mechanism of *SEMA4D*/*PlexinB1* in the bone marrow microenvironment such as bone marrow stromal cells and mesenchymal stem cells, so as to better understand the role of *SEMA4D*/*PlexinB1* in acute myeloid leukemia.

**Fig. 6:**
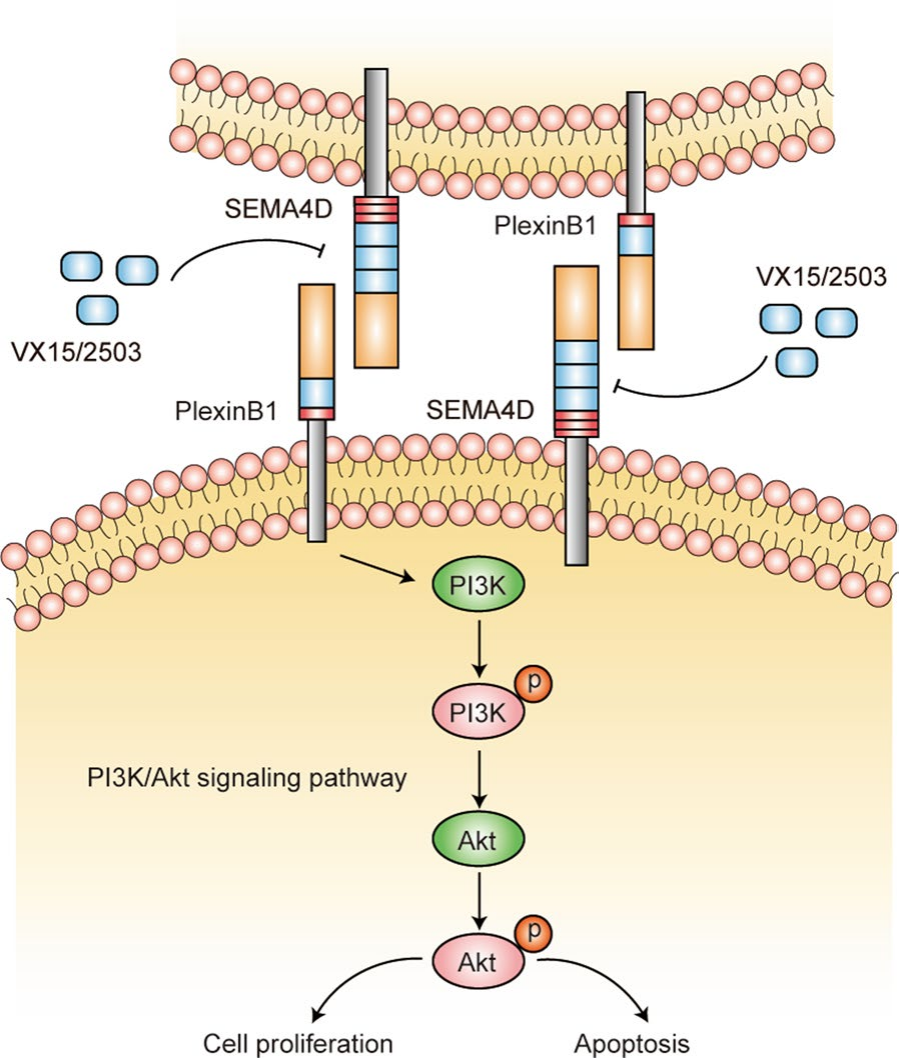
Schematic of proposed pathway depicting the role of SEMA4D/PlexinB1 in AML

In conclusion, we demonstrate the clinical significance of *SEMA4D* in human AML by showing that *SEMA4D*/*PlexinB1* promotes the progression of AML by activating the *PI3K*/*Akt* signaling pathway. A schematic diagram of a proposed pathway to describe the role of *SEMA4D*/*PlexinB1* in AML is shown in Fig. 6*SEMA4D* may serve as a novel target for diagnostically relevant biomarkers and combination therapy in AML patients.

## Data Availability

Publicly available datasets were analyzed in this study. This data can be found at the following hyperlinks: http://gepia.cancer-pku.cn/; http://www.cbioportal.org/.

## Abbreviations

AML: Acute Myeloid Leukemia
SEMA4D: Semaphorin 4D
PI3K: Phosphatidylinositide 3-kinases
AKT: Protein Kinase B
GEPIA: Gene Expression Profiling Interactive Analysis
IC50: Half maximal inhibitory concentration
